# The Role of Glutamine Synthetase (GS) and Glutamate Synthase (GOGAT) in the Improvement of Nitrogen Use Efficiency in Cereals

**DOI:** 10.3390/biom13121771

**Published:** 2023-12-10

**Authors:** Stefania Fortunato, Domenica Nigro, Cecilia Lasorella, Ilaria Marcotuli, Agata Gadaleta, Maria Concetta de Pinto

**Affiliations:** 1 Department of Biosciences, Biotechnology and Environment, University of Bari Aldo Moro, Via Orabona 4, 70125 Bari, Italy; stefania.fortunato@uniba.it (S.F.);; 2 Department of Soil, Plant and Food Sciences, University of Bari Aldo Moro, Via Orabona 4, 70125 Bari, Italy; domenica.nigro@uniba.it (D.N.); ilaria.marcotuli@uniba.it (I.M.)

**Keywords:** cereals, glutamate synthase, glutamine synthetase, nitrogen assimilation, nitrogen use efficiency, rice, maize, wheat

## Abstract

Cereals are the most broadly produced crops and represent the primary source of food worldwide. Nitrogen (N) is a critical mineral nutrient for plant growth and high yield, and the quality of cereal crops greatly depends on a suitable N supply. In the last decades, a massive use of N fertilizers has been achieved in the desire to have high yields of cereal crops, leading to damaging effects for the environment, ecosystems, and human health. To ensure agricultural sustainability and the required food source, many attempts have been made towards developing cereal crops with a more effective nitrogen use efficiency (NUE). NUE depends on N uptake, utilization, and lastly, combining the capability to assimilate N into carbon skeletons and remobilize the N assimilated. The glutamine synthetase (GS)/glutamate synthase (GOGAT) cycle represents a crucial metabolic step of N assimilation, regulating crop yield. In this review, the physiological and genetic studies on GS and GOGAT of the main cereal crops will be examined, giving emphasis on their implications in NUE.

## 1. Introduction

Cereals, in their broad category, are traditionally the most important crops produced and traded across the world for many different purposes [1]. Cereals, cultivated in large quantities, represent the primary source of food worldwide, providing most proteins and calories utilized by humans [2,3]. Due to the broad variety of derived foods, cereals are estimated to make available around 56% and 50% of caloric intake and protein needs [4]. Rice (*Oryza sativa*), wheat (*Triticum* ssp.), maize (*Zea mays*), and barley (*Hordeum vulgare*) are the most highly used crops for human nutrition, contributing to about 50% of the world’s food, both directly with the grain, or indirectly through livestock products [5]. FAO forecast reports indicate that the world’s population is expected to exceed 9 billion by 2050 [6], and this rise will require a 70% increase in food demand, mainly in cereals [7].

High yields of cereal crops strictly depend on a suitable supply of nitrogen (N), which is one of the most important mineral nutrients for plant growth. Indeed, N is a major constituent of fundamental compounds such as amino acids, proteins, nucleic acids, and chlorophylls. N deficiency can negatively affect photosynthetic and the reproductive capability of plants, reducing the yield in terms of biomass and grains, and can strongly affect resistance versus biotic and abiotic stressors. In cereals, N availability also influences the quality traits of the seeds, which have around 6% N in their storage protein reserves [2,8,9].

Since the yield of crops depend on N accessibility, in the last decades, a massive production and application of N fertilizers worldwide has been achieved. Currently, 122 million tons of N fertilizers are utilized worldwide in crops to reach desirable yields, and half of the total is used to fertilize maize, wheat, and rice [1,2]. Although N fertilization is the most important driving force for reaching yield potential in agriculture, the large use of these fertilizers may cause negative consequences for the environment, ecosystems, and human health [10,11]. Indeed, more than 50% of applied N fertilizers are not used up by crops and are wasted, causing a rise in soil acidification, emissions of gaseous ammonia into the atmosphere, and nitrate levels in water sources, with hazardous effects for human health [12,13]. Due to the negative effects on the environment and the cost of N fertilizers, the agronomic production model of improving yield only by raising N fertilization has become unsustainable [14,15]. Indeed, crop plants are frequently not effective in acquiring and using all the N supplied and accordingly, N fertilization does not inevitably lead to a proportional yield enhancement [16,17]. Despite the impressive boost in fertilizer application, the growth rate of crops has significantly slowed, and in developed countries is near to zero [18].

Cereals are described as one of the less efficient crops in terms of N use efficiency (NUE) [19]. Plant NUE can be defined as the ratio between the amount of N fertilizer applied and the amount of N removed with the harvest, or as the highest number of seeds or biomass obtained from a definite amount of N supplied [20]. NUE is the combination of N uptake efficiency (NUpE) and N utilization efficiency (NUtE). NUpE is described as the total amount of N acquired by the plants divided by the total N availability in the soil [21]. NUtE combines the plant’s capability to incorporate inorganic N into its carbon skeletons and remobilize the assimilated N from source-to-sink, and represents the portion of acquired N converted to grain or biomass [20,22].

In plants, N uptake, transport, assimilation, and remobilization are regulated by an intricate network of genes whose physiological and molecular mechanisms have been widely studied, especially in rice, maize and wheat [23,24,25]. However, gaining better knowledge of the intricate N metabolism, that could permit the enhancement of NUE in cereals, represents an important goal for achieving sustainable agriculture, leading to a decrease in dangerous environmental effects and supporting production to meet global demand [23,26,27,28,29]. One of the main targets of plant breeders is the generations of cultivars with high NUEs and trying to understand how genetic differences affect N uptake, utilization, and remobilization into seeds, especially under contrasting nitrogen regimens [30,31]. Broad research attempts have been dedicated to finding the genetic basis of NUE in crops, but success in achieving N use-efficient genotypes is limited since the NUE trait is very complex, being influenced by the genotype, environment, and N management [28,32,33].

The glutamine synthetase (GS)/glutamate synthase (GOGAT) cycle is principally involved in N assimilation and represents a crucial metabolic step in regulating NUtE and grain yield. In this review, we discuss the genetic and physiological findings on GS and GOGAT, highlighting their roles in NUE of the main cereal crops.

## 2. GS-GOGAT Cycle 

**Figure 1:**
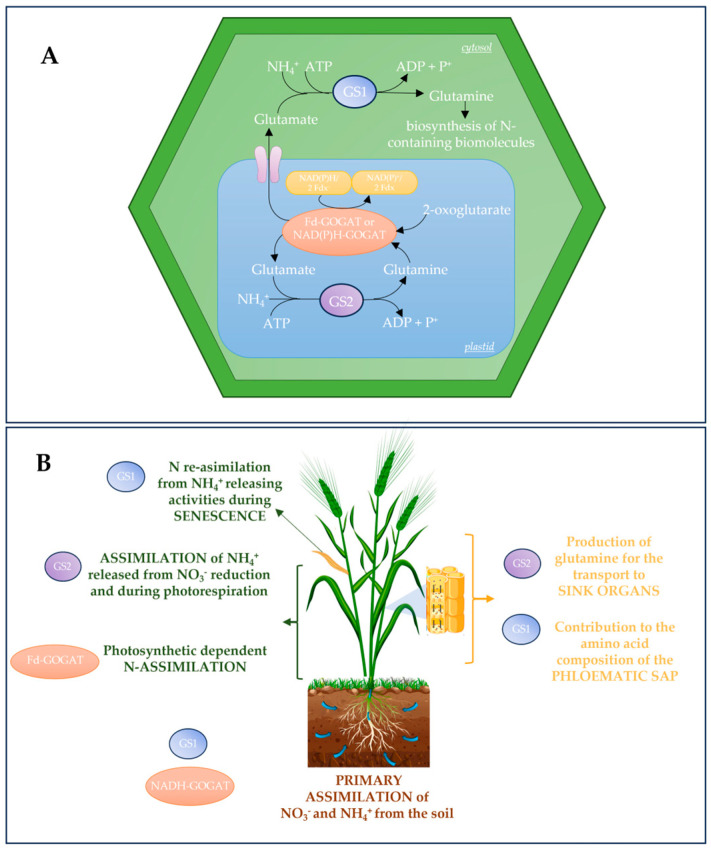
Cellular localization of the GS-GOGAT cycle and main roles of GS and GOGAT isoenzymes in plants. (**A**) Schematic representation of the GSGOGAT cycle in a plant cell. GS (GS1 localized in the cytosol and GS2 in the plastid) catalyzes the ATP-dependent condensation of ammonium (NH_4_^+^) and glutamate to form glutamine. GOGAT (NAD(P)H-GOGAT or Fd-GOGAT, both localized in plastid) transfers the amide amino group of glutamine to 2-oxoglutarate, allowing the synthesis of two molecules of glutamate: one used for biosynthesis of N-containing biomolecules, the second one becoming a substrate for GS to restart the GS/GOGAT cycle. (**B**) GS and GOGAT isoenzymes play different tasks depending on their localization. GS1 is involved in the primary N assimilation in the roots and in N remobilization to the grain during leaf senescence; GS1 also contributes to defining the aminoacidic composition of phloematic sap. GS2 plays a key role in NH_4_^+^ assimilation released during photorespiration and in glutamine synthesis for the transport to sink organs. NAD(P)H-GOGAT is involved in N primary assimilation, mainly in nonphotosynthetic tissues. Fd-GOGAT has an essential role in N assimilation relative to photosynthetic processes. Abbreviations: GS—glutamine synthetase; GOGAT—glutamine:2-oxoglutarate aminotransferase or glutamate synthase; N—nitrogen. Partially designed by Freepik.com; accessed on 12 October 2023.

Ammonium absorbed by the roots, generated by nitrate reduction, or released during amino acid catabolism or photorespiration is incorporated into glutamine and glutamate, respectively, by the action of two enzymes, glutamine synthetase and glutamate synthase, which operate jointly in the GS/GOGAT cycle (Figure 1). The glutamine and glutamate serve as N donors in the biosynthesis of other amino acids and amides [34,35].

The first step in the biosynthesis of organic N compounds is carried out by GS, the enzyme that catalyzes the ATP-dependent condensation of ammonium (NH_4_^+^) and glutamate to form glutamine [36,37]. Based on their different sizes and subcellular localizations, GS isoforms are distinguished as cytosolic (GS1) or chloroplastic (GS2) [36,38]. In most plants, except for wheat, a single gene exists for the chloroplastic GS2 isoforms. On the other hand, from three to five genes encode the cytosolic GS1 isoforms, suggesting a complex role for GS in several facets of plant N metabolism [39,40,41].

The cytosolic GS1 isoforms have a molecular weight that ranges from 38 to 40 kDa. Different GS1 isoforms may perform different tasks depending on their expression patterns and kinetic properties. GS1 plays a significant role in the primary assimilation of inorganic nitrogen derived from the soil as nitrate or NH_4_^+^ [42,43,44]. Furthermore, GS1 is involved in the N reassimilation from NH_4_^+^-releasing activities, like transamination of amino acids and phenylpropanoid, and protein degradation during senescence [37,45,46,47,48]. Since GS1 is the main isoform in the companion cells of phloem, it plays a major role in defining the amino acid composition of phloematic sap [49].

The chloroplastic GS2 isoform is larger than GS1, with a molecular weight ranging from 42 to 45 kDa. GS2 is mainly involved in the assimilation of NH_4_^+^ released from nitrate reduction in plastids and during photorespiration [50,51,52]. Plants deficient in GS2 are not able to survive under normal atmospheric conditions since they accumulate quite elevated amounts of NH_4_^+^ in the leaves [50]. The glutamine produced by the activity of GS2 in chloroplasts can be transported to sink organs or can be exchanged with other biochemical products by carboxylate transporters [53], affecting the production of other compounds such as proline, aspartate, or serine [54].

The expression of the different GS isoforms is highly regulated by light and N supply in specific tissues and developmental stages [55,56]. Moreover, GS activity can be regulated by posttranslational modifications like phosphorylation and 14-3-3 binding [57,58,59], as well as by redox changes and nitric oxide [60,61,62,63].

The reaction catalyzed by glutamate synthase, designated by the acronym GOGAT (glutamine:2-oxoglutarate aminotransferase), transfers the amide amino group of glutamine to 2-oxoglutarate, allowing for the synthesis of two molecules of glutamate: one used for the biosynthesis of N-containing biomolecules, such as other amino acids, nucleotides, and chlorophyll; the second one becoming a substrate for GS to restart the GS/GOGAT cycle (Figure 1) [64]. Glutamate synthase occurs in two distinct forms, the Fd-GOGAT and NADH-GOGAT, that use reduced ferredoxin (Fd) and nicotinamide adenine dinucleotide (NADH), respectively, as electron donors [64].

Fd-GOGAT is a monomeric protein with a molecular mass ranging from 145 kDa to 180 kDa, consisting of four distinct globular domains, namely the amidotransferase domain (GAT), the central domain, the flavin mononucleotide (FMN)-binding domain, and the C-terminal domain [65]. The GAT domain carries out the hydrolysis of glutamine, releasing the first molecule of glutamate. In the FMN domain, ammonia reacts with 2-oxoglutarate (2-OG) to form 2-iminoglutarate (2-IG), which is then reduced by the FMN cofactor. Ferredoxin reversibly binds a specific 26-amino acid loop in the 3Fe-4S cluster; this interaction induces a conformational change that allows glutamine to bind the cysteine in the active site, producing the second molecule of glutamate [66,67,68].

NADH-GOGAT is a protein with a molecular mass of 200 kDa, consisting of two subunits, namely α and β, with an FMN domain and 3Fe-4S cluster, respectively. In higher plants, the two subunits are linked by a 60-amino acid connector region. The α subunit carries out the glutamine amidotransferase reaction and the transport of electrons to 2-iminoglutarate mediated by FMN and the 3Fe-4S cluster, while the β subunit transfers electrons by NADPH to the α subunit [64].

The two GOGAT isoforms have specific roles in the different pathways of N metabolism. Both types of GOGAT are localized in the plastids, but they show distinct tissue specificities and biochemical properties. In leaves, nearly 97% of GOGAT activity is due to the Fd-dependent isoform localized mainly in mesophyll cells and to a lesser extent in bundle sheath cells, parenchyma cells of the fully expanded green leaf blades, and developing non-green leaf blades [66,69,70].

Fd-GOGAT is highly expressed in photosynthetic tissues, where it can directly utilize light energy as a supply of reductants [71]. Its activity is highly induced by light or by exogenously supplied sucrose, implying that its function is strongly correlated with secondary N assimilation and is dependent on photosynthetic processes [70]. In the last decades, in addition to N assimilation, some papers have reported Fd-GOGAT involvement in other physiological processes. In leaves, Fd-GOGAT has been associated with chlorophyll synthesis; Arabidopsis mutants deficient in Fd-GOGAT activity are chlorotic and show a conditional lethal phenotype when grown in high CO_2_ environments, proving that Fd-GOAGT has an essential role in growth [72,73]. In Arabidopsis and rice, Fd-GOGAT is reported to be targeted not only in the chloroplast but also in the mitochondria, where it interacts with serine hydroxymethyltransferase1 (SHMT1); this interaction underlines the role of Fd-GOGAT in the photorespiratory process [73,74].

NADH-GOGAT is expressed in the plastids of non-photosynthetic tissues, where the electrons necessary for reduction are provided by the pentose phosphate pathway [71]. NADH-GOGAT has been detected in the seeds, endosperm, roots nodules, apical meristem root primordial, epidermis, exodermis, and central cylinder of roots [66,67,75]. NADH isoforms at low levels are also expressed in leaves and vascular cells, where they represent only 3% of the total leaves’ GOGAT activity; nonetheless, it has been shown that Fd-GOGAT cannot compensate for this activity [70].

## 3. GS and GOGAT Isoforms in NUE of Cereals

Both GS1 and GS2, as well as NADH-GOGAT and Fd-GOAGT of different cereals have been proposed as key players in NUE, contributing to reach high crop yield or high grain protein content (GPC) [25,76].

In rice (*Oryza sativa*), the chloroplastic GS2 protein is coded by one gene (*OsGS2*), while three genes, designated as *OsGS1;1*, *OsGS1;2*, and *OsGS1;3* code the cytosolic GS1 protein (Table 1). *OsGS1;1* is expressed in all organs, with a high expression in the vascular tissue of mature leaves, playing a significant role in grain filling [75,77]. *OsGS1;2* transcripts have also been detected in all organs, with a higher expression in roots; this GS1 isoform plays a primary role in the assimilation of NH_4_^+^ taken up by the soil [78,79]. The *OsGS1;3* transcript is mainly present in spikelets, and it has a key role in grain ripening and germination [46,75,80]. *OsGS1;3* expression is regulated by the multifunctional regulator NF-YC12, which controls the accumulation of storage proteins in the endosperm [81].

The activities of the GS1 and GS2 enzymes in the flag leaves of rice are positively associated with the total GPC [91]. Nonetheless, in the grain filling stage, the total GS activity decreases, essentially due to the loss of GS2 [92], indicating that GS1 represents the principal contributor to total GS activity, participating in the development and quality of grains. The knockout of cytosolic *OsGS1;1*, which is localized predominantly in the vasculature of mature leaves, causes a drastic reduction in shoot growth and grain filling, along with serious metabolic imbalances [46]. Moreover, *GS1;1* RNAi transgenic rice show a drastic reduction in the panicle number and number of seeds per panicle [93]. Through a genome-wide association analysis, *OsGS1;1* has been identified as a NUE-associated gene. By alternative splicing, *OsGS1;1* generates two transcripts: *OsGS1;1a* and *OsGS1;1b*. In the elite haplotype of *OsGS1;1*, the expression of *OsGS1;1b* considerably increases GS activity. Moreover, *OsGS1;1b* overexpression enhances NUE, increasing grain dimension and reducing the content of amylose; thus, improving the quality of the rice grain through the regulation of sugar metabolism [94]. In the knockout of *OsGS1;1* mutants, the isogenes *OsGS1;2* and *OsGS1;3* do not compensate for the loss of *OsGS1;1*; this signifies a nonoverlapping and nonredundant role [46,95]. Interestingly, when leaf N is reallocated to the developing reproductive organs, the different GS isoenzymes work together to achieve the rice life cycle [93]. Also, knockout mutants of the *OsGS1;2* gene, which is expressed mainly in the surface cells of roots, show a drastic reduction in the active tiller number and panicle number [78], denoting its important role in the primary assimilation of NH_4_^+^ ions taken up by rice roots from the soil. The *OsGS1;1* isoform, also present to some extent in the roots, was unable to compensate for this function [78,79]. *OsGS1;2* expression has also been detected in leaves and stems [82]. *OsGS1;2* mutants show low levels of transcripts for carbon and N metabolism, causing metabolic disorder with a critical decline in lignin deposition and the development of axillary buds [79].

In rice, two genes, namely *OsNADH-GOGAT1* and *OsNADH-GOGAT2*, code for NADH-GOGAT, whereas Fd-GOGAT is coded by one gene [75]; in addition, the existence of a pseudogene for rice *Fd-GOGAT* has been reported [96]. *NADH-GOGAT1* is expressed in the root tips, endodermis, and exodermis [97], as well as in young leaf blades and grains during development [75,98]. In rice roots, the enzyme NADH-GOGAT1 performs a crucial role in the glutamate production for ammonium assimilation, and it is also essential in the development of active tillers in paddy fields [99]. The expression of *NADH-GOGAT2* and *Fd-GOGAT* in *nadh-gogat1* mutants does not differ from wild type plants, suggesting that the function of NADH-GOGAT1 in primary ammonium assimilation cannot be replaced by other GOGATs [99]. The *NADH-GOGAT2* gene is expressed mainly in completely expanded leaf blades and leaf sheaths. The knockout rice mutants lacking *NADH-GOGAT2* show a significant reduction in the spikelet number per panicle and a decline in yield and plant biomass, suggesting that NADH-GOGAT2 has a key role in the remobilization of leaf N to the panicles during senescence [100]. Moreover, knockout mutations of *NADH-GOGAT2* have proved to be unfavorable for seed production [101]. However, the insertion of Tos17 (transposon of *Oryza sativa* 17) in both *NADH-GOGAT1* and *NADH-GOGAT2* causes a significant increase in the free amino acid concentration and in protein content, indicating an improved nutritional quality of the grains [102]. Immunolocalization studies show that two distinct types of Fd-GOGAT, the leaf isoform Fd-GOGAT and root isoform Fd-GOGAT, are present in rice, even if the DNA sequence of the root isoform is not found in the rice genome database, and its function is still not clear [67,97,103,104]. The rice *OsFd-GOGAT* mutant *gogat1* shows only 33% of the total GOGAT enzyme activity in leaves exhibiting chlorosis under natural condition. This mutant has premature leaf senescence that facilitates N remobilization, leading to a significant increase in GPC [105]. Additionally, the altered carbon metabolism in the rice mutant *abc1*, defective for Fd-GOGAT1, indicates a pivotal role for Fd-GOGAT in modulating nitrogen assimilation and carbon/nitrogen balance [106].

In maize (*Zea mays*), there are five genes (*ZmGln11*, *ZmGln1;2*, *ZmGln1;3*, *ZmGln1;4*, and *ZmGln1;5*) coding for cytosolic GS1 and one gene (*ZmGln2*) encoding the chloroplastic GS2 [86,107]. *ZmGln2* is just expressed in the early stages of plant development, probably to reassimilate the ammonium released during photorespiration, which is at a low rate in the C4 plants [107]. The five cytosolic GS genes are differentially expressed in the roots, stems, and leaves (Table 1) [85,108]. The transcript of *ZmGln1;2* is abundant in the pedicel and pericarp and represents an important GS isoenzyme in developing kernels [83,84]. *ZmGln1;5* is expressed at a very low level in the leaves, roots, and stems [85,108]. *ZmGln1;3* and *ZmGln1;4* are highly expressed irrespective of leaf age and the level of N fertilization; however, an increase in *ZmGln1;4* expression occurs in older leaves. Both the enzymes ZmGln1;3 and ZmGln1;4 play a role in the correct development of cobs in relation to the kernel number and kernel size, respectively [86,109]. *ZmGln1;4* is expressed in bundle sheath cells and seems to be implicated in the ammonium reassimilation during protein degradation in senescing leaves [86,87]. In the *gln1.3* and *gln1.4* single mutants, as well as in *gln1.3*/*gln 1.4* double mutants, a significant reduction in GS activity occurrs. The knockout mutations of *ZmGln1;3* and *ZmGln1;4* result in a reduced kernel number and kernel size, respectively, and both phenotypes are evident in the *gln1.3*/*gln 1.4* double mutants. However, the production of shoot biomass does not change, indicating a specific effect on grain production [86,110].

In barley (*Hordeum vulgare*), one gene (*HvGS2*) encoding the chloroplastic GS2 and five genes (*HvGS1;1*, *HvGS1;2*, *HvGS1;3*, *HvGS1;4*, *HvGS1;5*) for cytosolic GS1 have been identified [88,111]. Transcripts of *HvGS1;1* are predominantly present in vascular tissues and play an important role in N transport and remobilization. *HvGS1;2* is expressed in the mesophyll cells of leaves and in the cortex and pericycle of roots, functioning in the primary N assimilation. *HvGS1;3* is mainly and specifically expressed in grains. However, under high NH_4_^+^ fertilization, *HvGS1;3* expression increases in the roots, suggesting a role in the defense against ammonium toxicity (Table 1) [88]. The analysis of four barley genotypes differing in NUEs showed that N-efficient genotypes had significantly higher GS and GOGAT activities in comparison with the other two genotypes with a relative N inefficiency [112].

In bread wheat (*Triticum aestivum*), three genes (*TaGS2a*, *TaGS2b*, and *TaGS2c*) encode the chloroplastic isoform of GS2 [40,113] and seven genes encode the three isoforms of GS1. TaGS1;1 is coded by *TaGS1a*, *TaGS1b*, and *TaGS1c*; *TaGS1;2* (also known as GSr) is coded by *TaGSr1* and *TaGSr2*; and *TaGS1;3* (also known as GSe) is coded by *GSe1* [40,76]. Three GS1 genes and one GS2 gene have also been identified in each subgenome of the bread wheat Chinese Spring [114].

The GS2 isoform of wheat along with its task in ammonia assimilation is localized principally in the leaf mesophyll, where it performs as a regulator at the carbon–nitrogen metabolic branch point, preserving the glutamine–glutamate pool in the chloroplast on the level of substrates [115]. TaGS1 isoforms have different roles in N assimilation (Table 1), showing distinct tissue localizations and different responses to N treatments. The transcripts of *TaGS1;1* and *TaGS1;2* are present in the perifascicular sheath cells and vascular cells, respectively [40,49]. *TaGS1;1* and *TaGS1;2* expression increases during leaf senescence, indicating a key role of these isoforms in ammonia assimilation during N remobilization to the grain [40]. Using specific antibodies, it has been shown that TaGS1;1 and TaGS1;3 accumulate in mesophyll cells, where they participate in cytoplasmic NH_4_^+^ assimilation, whereas TaGS1;2 is localized in the vascular tissues of leaves and roots, implying that it may have a role in N transport. An ammonium treatment inhibited *TaGS1;1* and stimulated *TaGS1;3* expression, suggesting that the GS1;3 isoform can act in alleviating NH_4_^+^ toxicity [90]. The localization and expression of TaGS isozymes has also been reported in grains with TaGS1;2 localized in the vascular bundle, TaGS1;2 and TaGS1;1 in the chalaza and placentochalaza, TaGS1;1 and TaGS1;3 in endosperm transfer cells, and TaGS1;3 and TaGS2 in the aleurone layer. This localization study attributed TaGS1;3 to a key role in gluten synthesis [89].

In wheat leaves, GS activity was found to positively correlate with the leaf N content, soluble proteins, and grain yield, but negatively correlate with leaf senescence [116,117,118]. The relationship between GS activity and the quantity of N remobilized from the upper part of the plant or from the flag leaf to the grain observed in five wheat cultivars with different NUEs, indicated that GS activity in the leaves might be used as an indicator of the N status of plants [116,117]. The analysis of GS in different tissues and phenological stages of ten durum wheat genotypes has shown that there is also a positive correlation among GS activity and expression (GS1 and GS2) and grain protein content (GPC). Furthermore, after N supplementation, while GS expression and activity increase in the roots of all genotypes, in leaves, the GS behavior changes in genotypes differing in GPC. Indeed, nitrogen treatments, without affecting soluble protein content, reduce GS of high GPC genotypes and do not affect GS in low GPC genotypes. These data highlight that the genetic differences between cultivars, rather than the N supply, cause differences in the GPC [119]. This is consistent with the analysis of fourteen UK and French wheat cultivars and two French advanced breeding lines, which show that NUE variability is primarily related to the differences in N-utilization efficiency [120]. A comparative analysis of two wheat cultivars showed that *GS1* and *GS2* are highly expressed postanthesis and preanthesis, respectively, in a N-efficient genotype compared to a N-inefficient genotype. Furthermore, the spatial and temporal distribution of the GS isozymes in source-sink organs during development contributes to the N-pool strength and affects the N flow. The cooperation of different GS isoenzymes in different organs promotes the strength of N flow and accelerates N transport to the grain, enhancing NUE [121].

The gene *Fd-GOGAT* is highly conserved among the three homoeologous hexaploid wheat genes and in durum wheat allelic variation is associated with GPC [122]. *NADH-GOGAT* also has three highly conserved homoeologous genes with *NADH-GOGAT-3A* and *NADH-GOGAT-3B* associated with a higher GPC in durum wheat [123,124].

## 4. Correlation of NUE Quantitative Trait Loci with GS and GOGAT

Developing cereal varieties and genotypes that are able to use the N available to the plant more efficiently is one of the main goals of modern breeding programs to support a sustainable agriculture. Nevertheless, a better understanding of the genetic variation of N utilization and the identification of the key genes involved is essential to achieve successful results. The identification of valuable candidate genes that affect a trait is of great importance to track them with functional markers or to eventually clone the favorable and superior alleles. Despite the genetic complexity of NUE, several studies on large population-based quantitative trait loci (QTLs) have been carried out on major cereals. Among the syntenic conserved regions of different cereal genomes identified through a meta-analysis, one of the conserved regions having a strong link to NUE contains both *GS* and *GOGAT* genes [125].

Many studies have shown the colocalization of *GS* and *GOGAT* genes with NUE and/or other related physiological and agronomical traits quantitative trait loci (Table 2).

In rice, three among the seven identified QTLs associated with cytosolic GS activity are colocated close to the QTLs for physiological and agronomical traits affected by N recycling, like the spikelet number and the panicle weight on the main stem. In addition, a structural gene for GS1 was mapped in the QTL region for one-spikelet weight, suggesting that GS1 could represent a key component of NUE and yield, having a role in grain development during senescence most likely due to its N export capacity. Moreover, the GS activity of a cytosolic GS1 colocalizes with the QTLs for N remobilization and grain size, affecting the spikelet number, soluble protein content, and panicle weight [126].

Similar research was carried out in maize, where the QTLs for leaf GS activity are coincident with the QTLs for yield, showing the putative role of GS in maize kernels’ yield. A positive correlation between the nitrate content, GS activity and yield has been found in maize recombinant inbred lines [128,130]. Interestingly, the *ZmGS1;4* locus was found to be coincident with a QTL for the thousand kernel weight trait, and the *ZmGS1;3* locus was coincident with two QTLs for the thousand kernel weight trait and yield [32,129].

In common wheat, the correlation of GS activity with QTLs for physiological and agronomic traits, such as the GPC, were linked to an improved NUE [131]. Large-effect QTLs for grain N percentage and total grain weight was associated with the *GS1* locus, but lower correlations were found with the loci for grain yield [118]. The QTL for total GS activity of flag leaves was positively colocalized with the QTLs for grain and stem N amounts, but smaller correlations were established with the loci for grain yield components. The QTL for GS activity colocalized to a *GS2* gene mapped on the chromosome 2A and to the *GSr* gene on the chromosome 4A [40].

Several investigations have focused on the identification of QTLs related to grain yield and grain protein content and their colocalizations with structural genes, confirming the colocalizations of QTLs related to grain yield and GPC with genes encoding for both the cytosolic GS1 isoform [118,131,136,137] and the plastidic GS2 one [131,137].

In durum wheat, numerous quantitative studies carried out either on biparental populations or wider genotype collections have found the involvement of *GS* and *GOGAT* candidate genes in the control of GPC [124,133,134,136]. Interestingly, the plastidic *GS2* and the cytosolic *GS1.3* have been associated with QTL for GPC in homoeologous regions to those reported in bread wheat on the 2B and 4A chromosomes, respectively [133,136]. The development of two distinct sets of heterogeneous inbred family (HIF)-based NILs segregating the *GS2* and *Fd-GOGAT* genes obtained from heterozygous lines at those loci confirm the previously identified GPC QTLs on the 2A and 2B chromosomes, and the involvement of these genes in GPC control [135]. Due to the high number of QTLs related to NUE, GPC, and yield mapped to the chromosomal regions containing *GS2* in both bread and durum wheat, it is plausible that these regions may be useful in breeding to obtain wheat varieties with improved agronomic performance and NUEs [118,124,133,135,138].

Less genetic studies are available about GOGAT involvement in NUE and GPC. In rice, the QTLs for physiological and agronomical traits affected by nitrogen recycling and associated with the QTL for cytosolic GS activity are colocalized with two QTLs for *NADH-GOGAT*. The QTL for NADH-GOGAT protein content colocalizes with the locus of the structural gene coding for GS1 in a region containing a QTL for one-spikelet weight [127]. In maize, the QTL harboring *NADH-GOGAT* colocalizes with the QTL controlling seedling root traits, strengthening the importance of the contribution of root morphology to NUE [139]. In wheat, the *NADH-GOGAT* gene located in the QTL on chromosomes 3A and 3B was found to be a major candidate for NUE; the homoeologous on chromosome 3A colocalizes with the QTLs of N uptake at anthesis and straw N concentration at maturity, while the homoeologous on chromosome 3B colocalizes with the QTLs of NUtE, GPC, and postanthesis absorption [140]. Interestingly, the NUE QTL is conserved at the same orthologous loci as the *GOGAT* gene on wheat chromosome 3B, rice chromosome 1, sorghum chromosome 3, and maize chromosomes 3 and 8 [125,140].

In durum wheat, the expression variation of the two homoeologous *NADH-GOGAT* genes was found to be associated with a higher GPC [123]. Moreover, a study on a collection of almost 240 durum wheat genotypes has identified specific allelic variants for both *Fd-GOGAT* and *NADH-GOGAT* associated with the GPC QTL on the 2A chromosome [124]. A genome-wide association analysis on the same collection allowed for the identification of fourteen candidate genes for QTLs related to nitrogen metabolism, among which one was *NADH-GOGAT* [134]. Allelic variations of *Fd-GOGAT* in durum wheat are also associated with GPC, demonstrating that it could be potentially useful in breeding programs according to the role of primary GOGAT activity in the earlier grain-filling stage [89,124]. Additionally, rice mutants defective for *Fd-GOGAT1* show an altered carbon–nitrogen balance, implying the pivotal role of Fd-GOGAT in growth and development by the modulation of N assimilation [106].

## 5. Transgenic Cereals Overexpressing GS or GOGAT

**Figure 2:**
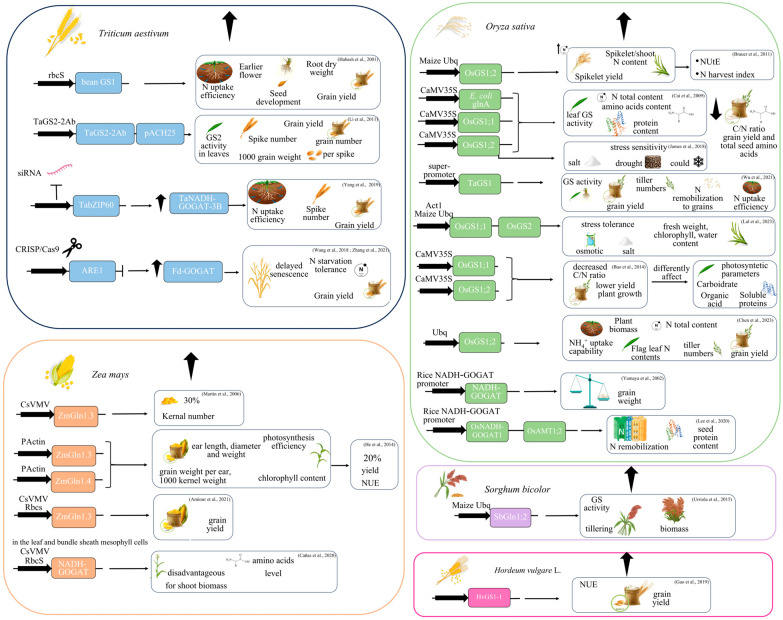
Effects of GS or GOGAT overexpression in transgenic cereals. Schematic representation of the constructs for the GS or GOGAT (black arrow indicates the promoter utilized) overexpression in transgenic cereals and related improved traits [35,86,92,141,142,143,144,146,147,148,150,151,152,153,157,158,159,160]. More details are given in the text. Abbreviations: ARE1—abnormal cytokinin response1 repressor1; Actin 1—Act1; CaMV—cauliflower mosaic virus; CsVMV—cassava vein mosaic virus; GS—glutamine synthetase; GOGAT—glutamate synthase; N—nitrogen; C—carbon; NUE—nitrogen use efficiency; RbcS—rubisco small subunit; Ubq—ubiquitin. Partially designed by Freepik.com; accessed on 24 October 2023.

The development of new cereal genotypes with a high yield under low N input is a fundamental approach to enhance agricultural sustainability. Given the crucial role of GS and GOGAT in regulating the yield and GPC in crops, many studies have tried to overexpress genes of the GS-GOGAT cycle in cereals to verify whether these transgenic plants could improve NUE (Figure 2).

The overexpression of GS has been conducted in several cereals, and even if not in all the cases, it has led to an improvement in NUE and/or seed yield.

In wheat (*Triticum aestivum*), the overexpression of the bean GS1 gene (*gln-α*) under the promoter of the rice rubisco small subunit (rbcS) improved N uptake efficiency with an increase in root dry weight and grain yield. The yield changes were attributable to an increase in grain N content and weight but not to grain number [92]. Transgenic plants also showed earlier flower and seed development but did not show an improvement in the shoot dry mass [92].

Since the decrease of total GS activity during grain filling is due to the loss of GS2 [92,117], the engineering of this gene has been more recently proposed to improve wheat NUE and grain yield [114]. In the mini-core collection of Chinese wheat varieties, the *TaGS2-2Ab* haplotype exhibited a considerable correlation with high shoot and root dry weight at seedling stage, and high grain N concentration and thousand grain weight [141]. *TaGS2-2Ab* codes for an enzyme with elevated GS activity compared with other haplotypes. The introduction of the allele *TaGS2-2Ab* with its own promoter in the variety Ji5265 of winter wheat substantially increased GS2 abundance and activity in leaves. Field experiments conducted for two consecutive years showed that these transgenic wheat plants improved the roots’ capability of N uptake before and after flowering, N remobilization to grains, and N harvest index. The transgenic lines showed improved spike numbers, grain numbers per spike, 1000-grain weight and grain yield compared to wild type plants when grown under both low- and high-N regimens. Moreover, the wheat plants overexpressing *TaGS2-2Ab* had a prolonged leaf functional extent, as shown by the chlorophyll content and net photosynthesis rate in flag leaves during the stage of grain filling [114].

An improvement in the grain number and yield has also been shown in maize plants overexpressing genes coding for GS1 [86,142]. *ZmGln1-3* encodes a GS isoform that gives adequate N to the growing ear to avoid kernel abortion, whereas *Gln1-4* codes for a GS isoform that reassimilates the ammonium released throughout leaf protein remobilization [87]. The constitutive overexpression of *ZmGln1;3* using the cassava vein mosaic virus (CsVMV) promoter caused a 30% rise in kernel number but no substantial differences in the shoot dry matter and NUE [86]. Likewise, maize plants overexpressing *Gln1-3* or *Gln1-4* grown in adequate N conditions showed an enhanced NUE and up to 20% of yield improvement compared with the WT [142]. The two transgenic lines showed increased yield-associated traits such as weight, diameter, length of ears, and grain weight per ear. Moreover, these transgenic plants had an increased chlorophyll content and photosynthesis efficiency, suggesting that the improved transfer of photosynthate from vegetative organs supports photosynthesis at the reproductive stage and slows down leaf senescence [142]. The overexpression of *Gln1-3* in the leaf and bundle sheath mesophyll cells of maize permitted an increase in kernel yield, which was mostly dependent upon the environmental conditions [143].

Different transgenic lines overexpressing *GS* genes have also been obtained in rice. The overexpression of the *OsGS1;2* isoform under the control of a maize ubiquitin (Ubq) promoter under high N conditions caused an increase in the NUtE with a higher N harvest index, expressed as the spikelet N content/shoot N content and spikelet yield compared to control plants [35]. On the other hand, these *OsGS1;2* overexpressing lines did not change the vegetative yield and shoot N content, suggesting that *OsGS1;2* overexpression enhanced N partitioning in rice during grain filling. However, under limiting N conditions, these plants did not show a better NUE, and consequently, it is unlikely that under field conditions they can take advantage of less N [35]. Cytosolic rice GS1 (*OsGS1;1* and *OsGS1;2*) and a *glnA* gene from *E. coli* under the control of the CaMV (cauliflower mosaic virus) 35S promoter were separately overexpressed in rice. All the transgenic lines displayed an improved metabolic level, with increased leaf GS activities and soluble protein contents and greater total N content and amino acid levels in the whole plants grown under both limiting and nonlimiting N regimes. Nevertheless, a decrease in both the grain yield production and total seed amino acids were observed in *GS*-overexpressing plants compared with wild-type plants. Finally only *GS1;2*-overexpressing plants showed a higher sensitivity to salt, drought, and cold stress, suggesting that improving the NUE can be accomplished by controlling specific GS isoenzymes [144]. The maintenance of a correct balance of carbohydrates and N metabolites, defined as the carbon/nitrogen (C/N) balance, is crucial for the control of plant growth, development, and yield [145]. A systematic study of the *OsGS1;1*- and *OsGS1;2*-overexpressing rice lines showed that due to N accumulation in the stem, these plants displayed a decreased C/N ratio with reduced plant growth and a lower yield. Moreover, the photosynthetic parameters, soluble proteins, and carbohydrates differed significantly in the *GS1;1*- and *GS1;2*-overexpressing plants. The metabolite profile and gene expression indicated that in *GS1;1-* and *GS1;2*-overexpressing plants, different changes occurred in the distinct sugars, organic acids, free amino acids and gene expression patterns, indicating the different tasks of the two *GS1* genes played in nitrogen metabolism. The alteration of the C/N balance and the difficulty to transport N from the stem to the leaf may account for the poor growth and yield observed in *GS1;1*- and *GS1;2*-overexpressing plants [146]. However, a very recent study indicated that rice plants constitutively overexpressing *OsGS1;2* under field conditions, with a naturally fluctuating environment, increased the plant biomass, tiller numbers, and N contents of flag leaf under different levels of N application. Moreover, these plants had a significant enhancement of physiological and agronomic parameters and an increase in grain yield under a low N treatment, suggesting that *OsGS1;2* overexpression could be a valuable strategy to combine an improved NUE, high grain yield, and reduction of N application. [147].

The overexpression of *TaGS1* in rice plants, which significantly increased the GS activity in leaves, junctions, and roots also led to increased tiller numbers and a higher grain yield. Moreover, the transgenic rice plants stimulated root capability of N acquirement and accumulation during growth and N remobilization to grains, giving a significant NUE enhancement [148].

Transgenic rice plants overexpressing both *GS1* and *GS2* under the control of rice actin 1 and maize ubiquitin promoters had an increase in fresh weight and became tolerant to N deficiency [149]. Moreover, the simultaneous overexpression of *OsGS1;1* and *OsGS2* isoforms in rice plants enhanced their tolerance to osmotic and salinity stress at the seedling stage. These transgenic lines retained considerably higher fresh weight, chlorophyll, and water content than the wild type, and showed less damage under stress. The grain-filling rates of these transgenic rice plants were also improved, leading to greater yields under adverse abiotic conditions [150]. Also, concurrent ectopic expression of *OsGS1;1* and *OsGS2* under the rice Actin1 and rice Actin2 promoters, respectively, improved NUE, permitting them to efficiently reassimilate the released ammonia. These transgenic rice plants demonstrated a better growth and productivity with an improved net photosynthetic efficiency [151].

In sorghum (*Sorghum bicolor*), the introduction of the *SbGln1;2* gene, encoding a cytosolic GS, under the control of the maize ubiquitin promoter caused an increase of up to 2.2-fold of GS activity and an enhancement of tillering and biomass production. Interestingly, the growth and development of *Gln1*-overexpressing plants were influenced not only by N availability, but also by other environmental factors. A substantial rise in the biomass and yield was observed in transgenic plants during the winter months, while in the spring, despite a small increase in the biomass, the seed yields decreased [152].

In barley (*Hordeum vulgare* L.) plants, the increase in GS1 activity, achieved through the cisgenic overexpression of an extra copy of native *HvGS1-1*, improved NUE and grain yield. This occurred under three distinct N regimens and two different concentrations of atmospheric CO_2_. Indeed, in transgenic barley plants, the additional ability for N assimilation, due to *GS1* overexpression, may help avoid the decline in GPC that occurs in wild-type plants when subjected to high atmospheric CO_2_ [153].

Since glutamate links carbon and N metabolism, GOGAT improvement appears as a potential approach for increasing yield [154]. However, even though a meta-QTL analysis highlighted the key role of this enzyme in NUE, only a few studies have been focused on the overexpression of GOGAT in cereals [20,125]. The overexpression of an *NADH-GOGAT* gene from japonica rice in an indica cultivar led to an increase in grain weight, suggesting that in rice, NADH-GOGAT is important for N utilization and grain filling [155]. On the contrary, *NADH-GOGAT* overexpression in maize plants was disadvantageous for shoot biomass production and did not significantly affect the kernel yield. The maize *NADH-GOGAT* overexpressing plants accumulated amino acids derived from glutamate and reduced the extent of carbohydrates altering the balance of carbon and N metabolism [156]. Interestingly, in rice, the combined overexpression of *OsNADH-GOGAT1* and rice ammonium transporter 1;2 (*OsAMT1;2*) enhanced NUE, leading to improved tolerance to N limitation and to a better N remobilization at the whole plant level. At sufficient N conditions, the transgenic plants showed high seed protein levels and an unaltered seed yield, while under low N both the seed protein levels and yield were increased [157]. Modifying transcription factors acting upstream of GOGAT also seems to be a successful approach for NUE enhancement. In wheat, an increase in NADH-GOGAT activity has been obtained by the knockdown of *TabZIP60* through RNA interference (RNAi). Indeed, TabZIP60 binds the ABRE element in the promoter of *TaNADH-GOGAT-3B* and negatively regulates its expression. The knockdown of TabZIP60 increased N uptake and the spike number, and improved grain yield under field conditions, indicating that the interaction of TabZIP60 and *TaNADH-GOGAT* was crucial in wheat NUE and growth [158]. Similarly, CRISPR/Cas9-mediated targeted mutagenesis of *ARE1* (abnormal cytokinin response1 repressor1), which is a suppressor of *Fd-GOGAT* [159], leads to tolerance to N starvation, delayed senescence, and increased grain yield in field condition [160].

## 6. Conclusions and Future Perspectives

The climate changing scenario is a major yield-limiting problem for cereal crops, since the consequent abiotic stresses restrict nutrient uptake, causing N deficiency. NUE improvement is assumed to be the most suitable approach to decrease costs and environmental issues produced by the high use of N fertilizers. In this context, the comprehension of the molecular mechanisms underlying N assimilation and N allocation to grains gives the chance to enhance the yield of cereals. GS and GOGAT have been shown to act as the main players in N assimilation, giving a major contribution to N utilization efficiency.

The study of the GS/GOGAT cycle also assumes a great importance since it is implicated in conferring tolerance to stress [161,162]. Moreover, GS is the target of phosphinothricin (PPT), an herbicide able to use the binding site of glutamate, and transgenic crops overexpressing GS for high PPT tolerance have been produced [144]. The products of the GS/GOGAT cycle, glutamine and glutamate, may also act as signal molecules participating in different pathways involved in plant growth and development, like the flowering time of rice [154,163]. It has been shown that the increase of glutamine can upregulate the expression of *Nhd1*, which in turn downregulates the expression and activity of Fd-GOGAT, showing a negative feedback regulatory pathway of N assimilation [163]. In *Arabidopsis thaliana*, the expression of *AtGLN1.3*, involved in N assimilation, is controlled by the regulating factor CCA1 of the circadian clock, whose expression is under the control of organic N [164]. In addition, the GS/GOGAT cycle also plays a critical role in the regulation of carbon and N balance, and GS activity needs ATP, while GOGAT uses C-skeletons in the form of 2-oxoglutarate and reductants like ferredoxin or NADH [165]. Consequently, the altered C/N balance, observed in transgenic rice overexpressing *GS1;1/GS1;2*, is responsible for the reduced growth and grain yield [146]. Therefore, since N assimilation must be finely controlled at the transcriptional and posttranslational levels, the factors regulating the GS/GOGAT cycle need to be deeply investigated.

NUE improvement by conventional breeding requires the identification of various gene pools; thus, the assessment of the natural variations of N assimilation in the germplasms of cereal crops, which exist in several agroecological environments, must be extensively explored to support the discovery of promising alleles of *GS* and *GOGAT*, which might be very beneficial for cereal NUE improvement. Since a moderate and slight variation occurs in the modern cereal cultivars, the source of appropriate genetic material should also be searched in ancient germplasms.

Genetic engineering may also significantly provide the prompt and accurate breeding of new cereal varieties with an improved NUE, representing a key approach for agricultural sustainability. Used bioengineering approaches to increase N assimilation consist of the overexpression of *GS* and *GOGAT* by their native or tissue-specific promoters. However, the manipulation of a single gene can induce negative consequences, due to the imbalance of metabolic intermediates or feedback control to retain homeostasis. These negative effects might be alleviated by stacking or pyramiding of the regulatory genes affecting N assimilation. Altering the GS and GOGAT activities by regulators, like transcription factors or partner proteins responsible for posttranslational modifications, might represent more promising strategies to achieve a suitable and stable degree of improved NUE. The identification of the genes able to affect the expression and activity of *GS* and *GOGAT* will help in the breeding of cereals with an improved NUE and yield through different methods like marker-assisted selection and genome editing. Until now, the utilization of genome editing methods has been modest, although the production of marker-free cereal crops with enhanced NUEs is nowadays feasible.

In conclusion, different approaches that consider the new, precise and refined techniques and strategies, like multiple allelic combinations and the CRISPR/Cas9-facilitated modification of key genes regulating N assimilation of cereal crops, have the potential to drastically decrease N fertilization and to improve grain yield for an increasingly sustainable agriculture.

## Figures and Tables

**Table 1:** Localization and main functions of the products of *GS1* genes in cereals.

Species	Genes	Principal Localization of Gene Products	Function	References
*Oryza sativa*	*OsGS1;1*	vascular tissue of mature leaves	grain filling	[75]
	*OsGS1;2*	leaves, stems, and roots	primary NH_4_^+^ assimilation	[78,79,82]
	*OsGS1;3*	spikelet	grain ripening and germination	[46,75]
*Zea mays*	*ZmGln1;2*	pedicel and pericarp	development of kernel	[83,84]
	*ZmGln1;3*	leaves, roots, and stems	development of cobs in relation to kernel number	[85]
	*ZmGln1;4*	leaves, roots, stems, and older leaves; bundle sheath cells	NH_4_^+^ reassimilation during protein degradation in senescing leaves; development of cobs in relation to kernel size	[86,87]
	*ZmGln1;5*	cortical tissue of seedling roots, vasculature of roots; seedling shoots and in stems	not reported	[85]
*Hordeum vulgare*	*HvGS1;1*	vascular tissues	N transport and remobilization	[88]
	*HvGS1;2*	mesophyll cells of leaves; cortex and pericycle of roots	primary N assimilation	[88]
	*HvGS1;3*	specifically expressed in grains; expressed in roots under high NH_4_^+^ fertilization	defense against NH_4_^+^ toxicity	[88]
*Triticum aestivum*	*TaGS1;1*	perifascicular sheath; mesophyll cells; chalaza and placentochalaza	cytoplasmic NH_4_^+^ assimilation	[40,89]
	*TaGS1;2/GSr*	vascular cells of leaves and roots; vascular bundle; chalaza and placentochalaza	N transport	[49,89]
	*TaGS1;3/GSe*	mesophyll cells; endosperm transfer cells; aleurone layer	cytoplasmic NH_4_^+^ assimilation; alleviating NH_4_^+^ toxicity; gluten synthesis	[89,90]

**Table 2:** *GS* and *GOGAT* genes colocalizing with NUE and other related physiological and agronomical quantitative trait loci (QTLs).

Species	Gene	Chromosome Localization	Colocalizing QTLs	References
*Oryza sativa*	*GS1*	2	Soluble protein content	[126,127]
		2	SPN, PNW	
		11	PNW, SPW, and RFD	
	*NADH-GOGAT*	1	Soluble protein content	[126,127]
		2	SPN, PNW	
		2	Soluble protein content, SPN, RFD, and RHD	
*Zea mays*	*Gln2* (cytosolic)	1	TKW, KN	[128,129]
	*Gln4* (cytosolic)	5	TKW, KN	
	*Gln1-3*	5	GY, TKW, leaf GS activity, NR activity, and leaf nitrate content	[130]
	*Gln1-3*	5	kernel yield and GS activity	[129]
*Triticum aestivum*	*GS2*	2	GS activity, soluble protein content/leaf	[118]
	*GS1*	6	TGW, grain N	
	*GSr*	4	GS activity, grain %N	
	*GSe*	4	GS activity	[40]
	*GSe*	4	GS activity	[131]
	*Fd-GOGAT*	2	GY, GN, and GPC	[132]
	*NADH-GOGAT*	3	NUE	[125]
*Triticum turgidum*	*GS2*	2	GPC	[124,133,134,135]
			GPD	[134]
	*Gse*	4	GPC	[133,136]
			GPD	[134]
	*GS1*	6	GPC	
	*GSr*	4	GPC	
	*Fd-GOGAT*	2	GPC	
	*NADH-GOGAT*	3	GPD	[134,135]

SPN: spikelet number on the main stem; PNW: panicle weight on the main stem; SPW: one spikelet weight; RFD: rate for full discoloration; RHD: rate for half discoloration; TKW: thousand kernel weight; KN: kernel number; GY: grain yield; GN: grain number; GPC: grain protein content; GPD: grain protein deviation; N: nitrogen; NUE: nitrogen efficiency use.

## Data Availability

No new data were created or analyzed in this study. Data sharing is not applicable to this article.
